# Pre-beta burst dynamics in Parkinson’s disease: distinguishing signal from artifact

**DOI:** 10.1002/mds.70317

**Published:** 2026-04-17

**Authors:** Bahman Abdi-Sargezeh, Tao Liu, Abhinav Sharma, Timothy Denison, Philip A. Starr, Simon Little, Vladimir Litvak, Ashwini Oswal

**Affiliations:** 1 MRC Centre of Research Excellence in Restorative Neural Dynamics, University of Oxford, Oxford, UK; 2 Nuffield Department of Clinical Neurosciences, University of Oxford, Oxford, UK; 3 Department of Neurosurgery, University of California San Francisco, San Francisco, USA; 4 Department of Neurology, University of California San Francisco, San Francisco, USA; 5 Wellcome Centre for Human Neuroimaging, University College London, London, UK

We thank Mirpour et al. for raising an important methodological consideration regarding the interpretation of pre-burst dynamics. Their argument—that threshold-crossing alignment of band-limited amplitude envelopes can produce an apparent pre-burst dip even in non-physiological signals—is well-taken. However, our analyses indicate that pre-burst dynamics in the parkinsonian subthalamic nucleus (STN) exhibit features that cannot be explained by analytic artifact alone.

We compared threshold-aligned burst envelopes in white noise and STN data for both prolonged and short burst durations (Figure 1A–B). White noise was used as a control signal to isolate analytical (filtering and threshold related) effects, as it lacks physiological autocorrelations and 1/f spectral structure (although core results are similar with pink noise). As noted by the authors, a pre-burst dip is present in noise and is similar for both burst categories. In contrast, prolonged bursts in STN data occur on the background of a higher baseline beta amplitude and exhibit a larger pre-burst dip. This duration-dependent dissociation—absent in white noise—indicates that the dip in real data carries information about the physiological properties of the forthcoming burst that go beyond what the alignment procedure alone generates. Interestingly, short duration bursts in neural data exhibit pre-burst profiles similar to those of bursts detected in noise, suggesting that these may reflect filtering-related physiological events rather than pathological oscillatory episodes [1,2].

We next examined the role of inter-burst temporal structure in shaping the pre-burst dip. Excluding bursts with inter-burst intervals (IBIs) <400 ms substantially attenuated the dip in real data (Figure 1C–D), indicating that elevated pre-burst beta power before prolonged bursts is largely driven by the offset of a preceding burst. In other words, the tail of a closely preceding burst elevates the baseline and deepens the apparent dip. This reflects a physiological feature—namely, the tight temporal clustering of pathological beta bursts. Consistent with this, neural data exhibited shorter IBIs and longer burst durations than white noise (Figure 1E–F), confirming that bursts in PD cluster more closely and persist longer than threshold crossings in noise.

To assess whether STN signals contain predictive features beyond those introduced by narrow-band filtering and envelope extraction, we repeated our analysis using broadband (4–90 Hz) data as classifier input while retaining the same burst annotations [3]. If predictability were solely driven by the detection pipeline, broadband features should not outperform noise. However, prediction performance was higher for neural data than for white noise (Figure 1G), indicating the presence of genuine predictive structure beyond the narrow-band envelope.

In summary, while threshold-crossing alignment contributes to the observed pre-burst dip, our analyses show that: (i) the dip in real data exhibits duration-dependent properties absent in white noise; (ii) it is strongly influenced by temporal clustering of bursts, reflecting genuine neural structure; and (iii) broadband prediction performance exceeds that in noise, indicating predictive features beyond the narrow-band envelope. Together, these findings suggest that pre-burst dynamics in parkinsonian STN recordings carry physiologically meaningful and clinically relevant information, even if partially shaped by the detection methodology.

## Figures and Tables

**Figure 1:**
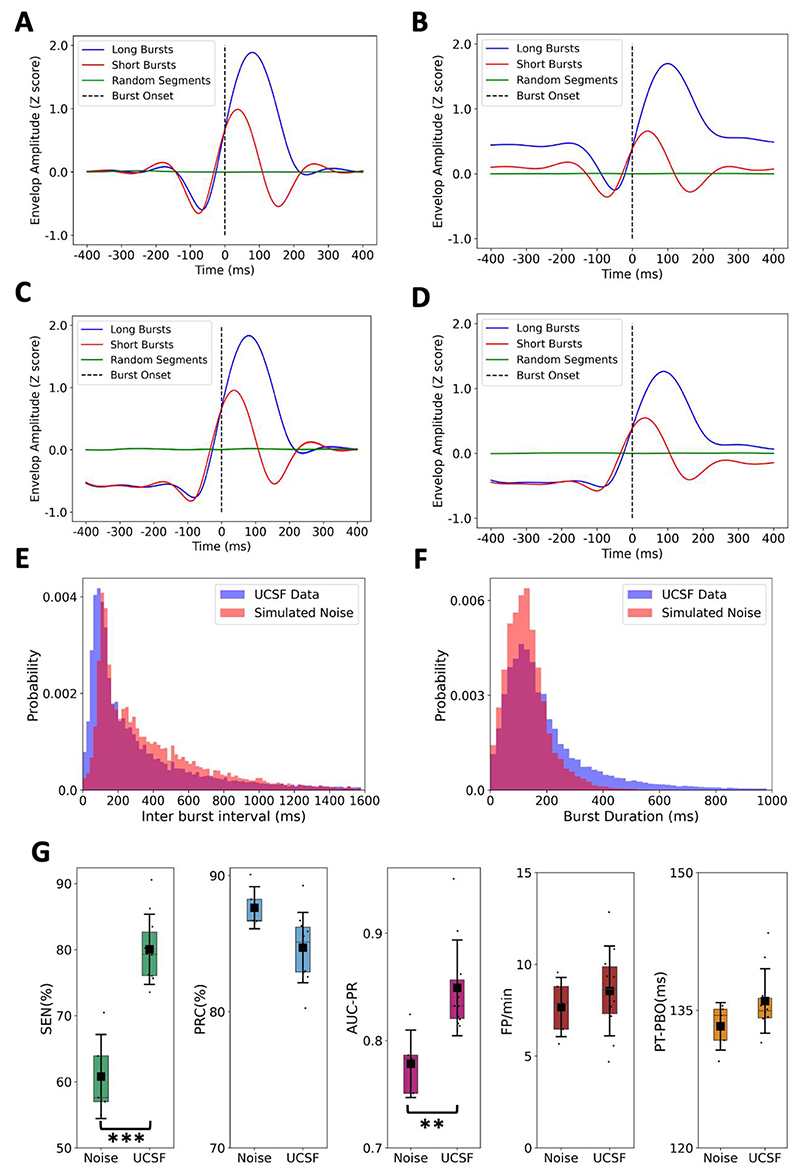
Pre-burst dynamics in STN recordings versus white noise. STN neural data are presented from 5 patients (10 hemispheres) with bilateral implants of the Summit RC+S neural interface (Medtronic), while white noise data are generated from 5 randomly selected seeds. **(A–B)** Z-score normalised, threshold-aligned burst envelopes for long (above median) and short (below median) duration bursts in white noise **(A)** and STN recordings **(B)**. In white noise, long and short bursts exhibit similar pre-burst profiles, whereas in STN data prolonged bursts show a deeper dip and higher baseline amplitude. **(C–D)** Threshold-aligned envelopes after exclusion of bursts with inter-burst intervals < 400 ms, for white noise **(C)** and real data **(D)**. The pre-burst dip is substantially attenuated, indicating that it is largely driven by preceding burst offsets. **(E–F)** Histograms of inter-burst intervals **(E)** and burst durations **(F)** for white noise and real data, demonstrating distinct temporal statistics. **(G)** Burst prediction performance is shown for the sliding window approach described in [3], using causally filtered broadband data (4-90 Hz) as input to the neural network. Five burst prediction performance metrics were compared for neural data and white noise—sensitivity (SEN), precision (PRC), area under precision-recall curve (AUC-PR), the rate of false positive predictions (FP/min) and the mean prediction time prior to burst occurrence (PT-PBO). Of these, only differences in SEN (Mann-Whitney U: U = 0.0, Holm-adjusted p = 0.0033***) and AUC-PR (Mann-Whitney U: U = 4.0, Holm-adjusted p = 0.032**) were significantly different and greater for neural data than for white noise.
